# Fungal Phytase Increased Ileal and Total Tract Digestibility of Phosphorus of Cold-Pressed Canola Cake and Canola Meal Diets in Growing Pigs

**DOI:** 10.3390/ani14233485

**Published:** 2024-12-02

**Authors:** Nestor Arce, Li Fang Wang, Adriana Morales, Miguel Cervantes, Ruurd T. Zijlstra

**Affiliations:** 1 Department of Agricultural, Food and Nutritional Science, University of Alberta, Edmonton, AB T6G 2P5, Canada; nestor-arce@hotmail.com (N.A.); lifang1@ualberta.ca (L.F.W.); 2 Instituto de Ciencias Agrícolas, Universidad Autónoma de Baja California, Mexicali 21100, Mexico; adriana_morales@uabc.edu.mx (A.M.); miguel_cervantes@uabc.edu.mx (M.C.)

**Keywords:** canola cake, canola meal, digestibility, phosphorus, phytase, pig

## Abstract

The co-product canola meal contains little fat and has been used in swine production as a protein source for several decades. More recently, locally produced canola cake has also become available that contains important quantities of residual oil. Both canola co-products contain a considerable quantity of phosphorus (P) with low availability. Phytase supplementation can increase P digestibility in canola meal but its efficacy remains unclear for canola cake containing high residual oil. To explore whether phytase supplementation can increase digestibility of P and other nutrients in canola cake, four diets containing 500 g of locally produced cold-pressed canola cake or canola meal, with or without supplemental phytase (Ronozyme HiPhos; *Aspergillus oryzae* origin)/kg, were fed to seven pigs (initial body weight; 55.9 ± 3.8 kg) in four 9-day periods following a 4 × 4 Latin square plus a 4 × 3 Youden square. Phytase supplementation increased phytase activity from below the detection limit to reach a minimum of 1000 phytase units/kg in the canola meal and cake diets; respectively. Phytase supplementation increased (*p* < 0.05) coefficients of ileal and total tract digestibility of P by 60% and 46% for canola meal and 76% and 57% for canola cake, respectively, but did not affect the digestibility of crude protein or gross energy. Ileal or total tract digestibility of P did not differ between the canola cake and canola meal diets in growing-finishing pigs. In conclusion, fungal phytase is effective in increasing the P digestibility of canola cake similar to in canola meal fed to growing-finishing pigs. The similar ileal or total tract digestibility of P in canola meal and cake diets either with or without phytase supplementation indicated that the residual oil in canola cake did not affect P digestion and did not affect the efficacy of phytase in increasing P digestibility.

## Simple Summary

Plant-origin phosphorus bound as phytate is difficult for pigs to use due to a lack of endogenous phytase in the gut and a lack of intrinsic phytase in feedstuffs such as in canola co-products. A lack of phytate degradation causes phosphorus excretion in manure. Dietary inclusion of phytase increases phosphorus digestion in canola meal that is low in fat; however, whether phytase can increase phosphorus digestion similarly for canola cake containing 20% residual oil remains unclear. We included 0.1 g phytase/kg in either canola meal or canola cake diets that increased phytase activity to above 1000 phytase units/kg compared to nil in two diets without phytase. We fed the diets to seven growing-finishing pigs over four 9-day periods. Phytase supplementation increased ileal and total tract digestibility of phosphorus by 60% and 46% for canola meal and 76% and 57% for canola cake, respectively, but did not affect protein or energy digestibility. In conclusion, phytase supplementation increased phosphorus digestibility for canola cake to a similar extent as for canola meal in growing-finishing pigs. Phytase supplementation may thus reduce phosphorus excretion in pigs fed diets containing low-fat or high-fat canola co-products in pigs.

## 1. Introduction

Canola, also known as double-zero rapeseed, is grown in temperate climates. Its annual production is 75 million metric tons globally, notably 20.3 million metric tons in Canada and 25.5 million metric tons in the European Union [1]. In Canada, solvent extraction of canola seed generates 5.4 million metric tons of canola meal annually [2]. Canola meal is used as an alternative protein source to soybean meal (SBM) in swine production [3]. Dietary inclusion of 200 g canola meal/kg to replace SBM did not affect feed intake or growth in weaned pigs [4]. Other canola co-products are also available for pig feeding [5]. For example, pressing canola seed without steam conditioning and solvent extraction generates canola cake, which contains more residual oil than canola meal, resulting in a greater net energy value [6,7,8]. Canola cake is thus an attractive feedstuff for swine [9].

Canola meal or cake contains 10–14 g phosphorus (P)/kg [7,10]. Whether the extent of P digestibility is similar between canola cake and meal in pigs remains unclear. For canola meal, 36–76% of P is bound as phytate with low bioavailability (21%) due to low intrinsic phytase activity in canola meal (5 phytase units [FTU]/kg) and a lack of endogenous phytase in the upper intestinal tract of pigs [11,12,13]. Such low availability of P combined with high dietary inclusion of canola co-products has led to interest in methods to increase P digestibility and reduce inorganic P supplementation to reduce feed cost and P excretion in manure [14]. Dietary supplementation of phytase can increase P digestibility of canola meal in pigs [15,16]. For co-products containing more residual oil than canola meal, for example, 00-rapeseed expeller, dietary supplementation of microbial phytase increased P digestibility [17]. In vitro, rapeseed cake soaked with phytase had increased phytase activity [18]. However, whether phytase supplementation can increase the P digestibility of canola cake in pigs has not been reported. Finally, phytase effects on protein digestibility in canola meal were not consistent in growing pigs and require further investigation [19].

The null hypotheses of the present study were that phytase supplementation in a diet containing canola cake would not alter crude protein (CP), gross energy (GE) and P digestibility and that the extent of change in nutrient digestibility would not differ between canola cake and canola meal diets in growing-finishing pigs. The objective was to determine and compare the effect of phytase supplementation on the coefficients of apparent ileal digestibility (CAID) and apparent total tract digestibility (CATTD) of CP, GE and P in a canola cake and canola meal diets fed to growing-finishing pigs.

## 2. Materials and Methods

The animal procedures for the study were reviewed and animal use was approved by the University of Alberta Animal Care and Use Committee for Livestock and followed principles established by the Canadian Council on Animal Care [20]. The study was conducted at the Swine Research and Technology Centre of the University of Alberta.

### 2.1. Experimental Diets

The solvent-extracted canola meal (Bunge Canada, Fort Saskatchewan, AB, Canada) and cold-pressed canola cake (Cansource Biofuels, Mayerthorpe, AB, Canada) originating from *Brassica Napus* canola seed were included at 500 g/kg in corn starch-based diets (Table 1). A 6-phytase enzyme (Ronozyme HiPhos, DSM Nutritional Products, Basel, Switzerland) derived from the fungus *Aspergillus oryzae* was included at 0.1 g/kg in the diets to provide a minimum of 1000 FTU/kg of diet. Diets were fortified with vitamin and mineral premixes to meet or exceed requirements [21]. Chromic oxide was included at 5 g/kg as an indigestible marker.

### 2.2. Experimental Design and Procedure

Seven crossbred barrows [initial body weight (BW), 55.9 ± 3.8 kg] were used in the study. Pigs had been surgically fitted with a simple T-cannula at the distal ileum, approximately 5 cm anterior to the ileocecal sphincter [22,23]. Seven pigs were fed the four diets in four 9-day periods in a 4 × 4 Latin square plus a 4 × 3 Youden square to provide seven observations per dietary treatment. Pigs were housed in raised individual metabolism pens that allowed freedom of movement (1.2 m wide, 1.4 m long and 0.94 m high). Pens were equipped with a stainless-steel self-feeder attached to the front of the pen and a cup drinker next to the feeder. Walls were made of polyvinylchloride with windows and the flooring was plastic-covered woven wire mesh. The test room temperature was maintained at 22.0 ± 2.5 °C. Each 9-day experimental period consisted of a 5-day adaptation to the experimental diets, followed sequentially by a 2-day collection of feces and a 2-day collection of ileal digesta. Pigs were fed diets at 2.5 times the maintenance requirement for DE (2.5 × 110 kcal of DE/kg BW^0.75^; [21]) in 2 equal meals at 0800 and 1500 and had free access to water throughout the experiment. Feces were collected using plastic bags snapped between a leather and a Velcro ring attached to the skin around the anus using medical adhesive [24]. Digesta samples were collected for 2 days from 0800 to 1800 using plastic bags containing 15 mL of 50 g formic acid/kg that were attached to the opened barrel of the cannula with a rubber band. Bags were replaced as soon as filled or after 20 min [25]. Collected feces and digesta were pooled for each pig in each experimental period and frozen at −20 °C. Upon completion of the trial, feces and digesta were thawed, homogenized, subsampled and freeze-dried.

### 2.3. Sample Preparation and Chemical Analyses

The diets and lyophilized feces and digesta were ground through a 1 mm screen in a centrifugal mill (model ZM 200; Retsch GmbH, Haan, Germany). Ingredients, diets, digesta and fecal samples were analyzed for dry matter (DM; method 930.15; [26]), CP (method 990.03; N × 6.25) using a combustion analyzer (model CNS-2000; Leco Corp., St. Joseph, MI, USA) and GE using an adiabatic bomb calorimeter (model 5003, Ika-Werke GMBH & Co. KG, Staufen, Germany) with benzoic acid as a standard. Ingredients were analyzed for crude fat (method 920.39A) as per AOAC [26]. Chromic oxide in diets, digesta and fecal samples was determined using spectrophotometry (model 80-2097-62, KBUltraspec III, Pharmacia, Cambridge, UK) at 440 nm after ashing at 450 °C overnight [27]. Ingredients, diets, ileal digesta and fecal samples were analyzed for P (method 946.06; [26]) by ashing (600 °C overnight) followed by digestion and then using spectrophotometry (model SPECTRAmax PLUS 384, Molecular Devices, San Jose, CA, USA) at 400 nm. Enzyme activity in the test diets was analyzed at the Analytical Research Center (DSM Nutritional Products Ltd., Kaiseraugst, Switzerland). One FTU is defined as the quantity of phytase that liberated 1 µM of ortho-phosphate per min from 5.1 mM Na-phytate at pH 5.5 and at 37 °C.

### 2.4. Calculations

The CAID and CATTD of components in the diet were calculated using data from duplicate chemical analyses and using the index method with the following equation [28]:

CAID or CATTD = 1 − (concentration of Cr_2_O_3_ in feed × concentration of component in feces or digesta)/(concentration of Cr_2_O_3_ in feces or digesta × concentration of component in feed).

The coefficient of apparent hindgut fermentation (CAHF) of components was calculated with the equation [29]:

CAHF = CATTD − CAID

### 2.5. Statistical Analyses

Data were analyzed using the GLIMMIX procedure of SAS v9.4 [30]. ‘Pig’ was the experimental unit. Normality and homogeneity of variance of the residual of each variable were confirmed using the UNIVARIATE procedure with the ‘Normal’ option and GLM procedure with the ‘Hovtest = Levene’ option, respectively. Carryover effect and outliers were checked prior to statistical analyses. Digestibility coefficients were analyzed as a 2 × 2 factorial arrangement. In the statistical model, co-product, phytase and their interactions were the fixed effects, whereas square, pig nested in square and period nested in square were the random factors. To test hypotheses, *p* < 0.05 was considered significant.

## 3. Results

For the experimental diets, the canola meal diets contained more CP but had lower GE values than the canola cake diets (Table 1). The canola meal and cake diets contained similar levels of P, and the targeted phytase supplementation was reached based on the analyzed dietary phytase activity. For the two canola co-products, canola cake contained 167 g/kg more fat and 34 g/kg less CP but had a 3.7 MJ/kg greater GE value than canola meal (Table 2). Both canola co-products contained similar total P.

The CAID values of DM, GE and CP were greater (*p* < 0.01; Table 3) for canola cake diets than for canola meal diets and were not affected by dietary phytase supplementation. The CAID of P did not differ between canola meal and cake diets. Phytase supplementation increased (*p* < 0.01) the CAID of P by 0.21 for the canola cake diet and by 0.16 for the canola meal diet.

The CATTD values of DM, GE, and CP were greater (*p* < 0.01; Table 4) for canola cake diets than for canola meal diets and were not affected by dietary phytase supplementation. The CATTD of P did not differ between canola meal and cake diets. Phytase supplementation increased (*p* < 0.01) CATTD of P by 0.18 for the canola cake diet and by 0.16 for the canola meal diet.

The CAHF values of DM, GE, CP and P were greater (*p* < 0.05; Table 5) for canola meal than canola cake diets. Dietary phytase supplementation did not affect the CAHF values of DM, GE, CP and P.

## 4. Discussion

### 4.1. Chemical Composition of Canola Co-Products

In the present study, the CP content of canola meal was in the reported range [4,31,32] but was greater than that in canola cake. The lower CP in canola cake can be explained by the dilution effect of the residual oil in canola cake [9]. Indeed, canola cake has a greater GE value than canola meal. Unlike solvent extraction, which depletes all the oil in canola meal, canola cake was produced without solvent extraction, thereby only removing part of the oil [33]. With phospholipids in the form of gums added back to the canola meal, the crude fat content of the canola meal is low (27–40 g/kg) [4,32,33]. Conversely, canola cake contains 96–242 g/kg crude fat depending on processing conditions [7,34].

In the present study, canola meal and cake contained around 10 g P/kg, similar to reported values [4,7,9,32]. Similarly, with the dilution effect of residual oil on CP content, canola cake contained 10% less P than canola meal. In the present study, phytase activity was below the detection limit in both canola co-product diets without phytase supplementation, indicating that intrinsic phytase activity in canola meal and cake was low, likely similar to the 5 FTU/kg phytase activity in Australian canola meal [12]. Considering canola meal or cake contains three times more P than cereal grains [12], high dietary inclusion of canola co-products without strategies to increase P utilization would raise P excretion.

### 4.2. Phytase and Ileal Digestibility of Nutrients and Energy

In the present study, canola cake diets had a greater CAID of DM, which was consistent with their greater CAID of CP and other energy-yielding nutrients. Although phytase supplementation increased the CAID of P of the canola meal and cake diets, the amount of P in the canola meal or cake diets was insufficient to alter the CAID of DM.

The CAID of GE was 0.08 greater for the canola cake than canola meal diets and matched the change in the CAID of DM. However, the magnitude of increase in the CAID of GE was lower than 0.135 for canola cake containing 202 g crude fat/kg compared to canola expeller containing 105 g crude fat/kg in growing pigs [9]. The greater CAID of GE in canola cake diets could be explained by the greater CAID of CP in canola cake and its greater residual oil that has greater ileal digestibility than other energy-yielding nutrients [34]. The lack of phytase supplementation effect on the CAID of GE in the present study indicated that phytase did not increase the CAID of energy-yielding nutrients in canola meal or cake, e.g., protein, fiber, or fat.

In the present study, the CAID of CP was greater in the canola cake than canola meal diets, similar to the greater CAID of CP in 00-rapeseed expeller than 00-rapeseed or canola meal in growing pigs [35]. Canola co-product was the sole dietary N source in the present study; thus, the CAID of CP in the diets can be compared to reported values for canola meal and expeller in growing pigs [31]. Dietary fat may increase the CAID of CP, because fat may reduce gastric emptying and digesta passage rate due to greater digesta viscosity in pigs as observed in broilers, thereby increasing retention time for nutrient digestion [36,37]. Increasing the dietary inclusion of canola oil but not canola cake did increase the CAID of CP [34]. In the present study, phytase supplementation did not increase the CAID of CP in the canola cake or meal diets. However, phytase effects on the CAID of CP are not consistent in growing pigs [19]. For example, phytase did not affect the CAID of CP in SBM in growing pigs and canola meal in finishing pigs [38,39]. Although phytase may increase the CAID of most dietary amino acids based on analyzed data from 28 studies, phytase effects remain inconclusive on increasing the CAID of CP in canola co-products in swine [40,41].

Without phytase supplementation, canola co-product diets had a low CAID of P, similar to the CAID of P at 0.23 in canola meal in growing pigs [42]. The low CAID of P is due to the low intrinsic phytase activity in canola co-products and a lack of endogenous phytase in the stomach and small intestine of pigs [11,13]. Considering 10 g inorganic phosphate/kg in diets that have greater P digestibility [43,44], the CAID of P in canola meal and cake is likely lower. The low CAID of P in the tested diets could be partly due to the endogenous P in pigs and affected by dietary P levels [42,45]. Phytase supplementation increased the CAID of P in both canola co-product diets, indicating that phytase degraded phytate in canola meal and cake in the stomach and proximal small intestine with favorable pH because supplemental phytase activity faded at the ileum of pigs [46,47]. The magnitude of the increase in CAID of P due to phytase supplementation was similar between canola co-product diets and SBM in growing pigs [38].

### 4.3. Phytase and Total Tract Digestibility of Nutrients and Energy

Without phytase supplementation, the CATTD values of DM and GE were greater in the canola cake than canola meal diet, similar to the greater CATTD of GE seen in rapeseed expeller than in rapeseed meal or canola meal [48,49], and the greater CATTD of GE seen in canola cake than in canola expeller [9]. The difference in the CATTD of GE was smaller than in the CAID of GE between canola cake and meal diets. This change was supported by greater hindgut fermentation of CP and GE in canola meal than in canola cake. Due to the lower CAID values of DM and CP in the canola meal diets, more undigested residue entered the hindgut for canola meal than canola cake diets.

In the present study, phytase supplementation did not increase the CATTD values of DM and GE, indicating that phytase had limited ability to liberate energy-yielding nutrients in canola co-products. Similarly, microbial phytase did not increase the CATTD of GE in diets containing cereal grains, SBM, or rapeseed meal in weaned or growing pigs [50,51,52]. The phytase effect on energy digestibility has been inconsistent. For example, fungal phytase increased the CATTD of GE in diets with low P levels but not with normal P levels [53]. Similarly, phytase increased the CATTD of GE in pelleted but not mash diets in weaned pigs [54].

In the present study, the CATTD of CP was greater for the canola cake than canola meal diets regardless of phytase supplementation, in contrast to the equal values of the CATTD of CP between low-fat canola meal and high-fat canola expeller [31]. Whether residual fat in canola co-products affects the CATTD of CP in pigs remains thus inconclusive. The greater CATTD than CAID of CP in diets indicated substantial undigested protein entered the hindgut as a substrate for microbial fermentation that may provide energy for the host [55].

Phytase supplementation had little effect on the CATTD of CP in pigs. In the present study, phytase did not increase the CATTD or CAHF of CP in canola meal or cake diets, indicating that phytase did not liberate protein from canola meal or cake during hindgut fermentation. Likewise, phytase did not alter the CATTD of CP of diets based on wheat and SBM in sows or on corn and SBM or barley and field pea in growing pigs [50,56,57,58].

Without phytase supplementation, the CATTD of P in canola meal and cake diets was low, similar to the 0.28–0.33 reported for the CATTD of P in canola meal in growing pigs [59]. The CAHF of P at 0.04–0.10 for canola meal and cake diets without phytase supplementation indicated further breakdown of phytate by microbial phytase and absorption of P in the hindgut [47,60,61]). Without exogenous phytase, phytase activity was negligible in the upper gut of pigs, but sharply increased in the colon, allowing breakdown of phytate [47].

Phytase supplementation increased the CATTD of P in the present study, indicating that fungal phytase is effective for increasing P digestibility in canola meal and cake and reduce fecal P excretion. Previously, microbial phytase increased the CATTD of P in canola meal in growing pigs [59,62,63]. However, the increased CATTD of P was mostly due to the increased CAID of P for the canola cake diet in the present study as evidenced by the CAHF of P at 0.04 in the canola cake diet and lack of phytase effect on the CAHF of P. Dietary supplementation of phytase may reduce microbial phytase activity in the colon of pigs [47]. The phytase effect on P digestibility of canola meal and cake in the present study was similar to the increased CATTD of P in 00-rapeseed expeller, canola meal, and 00-rapeseed meal [17]. Although increasing dietary fat increased P degradation in poultry due to increased mean retention time [64], the higher levels of crude fat in the canola cake than canola meal diets did not increase the CATTD of P in the present study.

## 5. Conclusions

Supplementation of fungal phytase in diets increased ileal and total tract digestibility of P but not of crude protein or gross energy in diets containing cold-pressed canola cake or solvent-extracted canola meal in growing-finishing pigs. The similar CATTD of P in the canola meal and cake diets, either with or without phytase supplementation, indicated that the residual oil in the canola cake did not affect P digestion and did not affect the efficacy of phytase for increasing P digestibility. However, an effect due to the 9% difference in crude protein content between the canola meal and canola protein diets on the measurements cannot be excluded.

## Figures and Tables

**Table 1:** Ingredient composition, analyzed nutrient content (g/kg), gross energy (GE) value and calculated crude fat content of the experimental diets (as fed).

Ingredient	Canola Meal		Canola Cake	
	Without Phytase	With Phytase	Without Phytase	With Phytase
*B*. *napus* canola meal	500.0	500.0	–	–
*B*. *napus* canola cake	–	–	500.0	500.0
Corn starch ^1^	400.1	400.0	400.1	400.0
Dextrose ^2^	51.7	51.7	51.7	51.7
Canola oil	15.2	15.2	15.2	15.2
Mono/dicalcium phosphate	10.0	10.0	10.0	10.0
Limestone	4.0	4.0	4.0	4.0
Salt	4.0	4.0	4.0	4.0
Vitamin premix ^3^	5.0	5.0	5.0	5.0
Mineral premix ^4^	5.0	5.0	5.0	5.0
Cr_2_O_3_	5.0	5.0	5.0	5.0
Phytase ^5^	–	0.1	–	0.1
Analyzed nutrient content				
Moisture	81.3	87.6	77.7	72.6
Crude protein	224.8	230.6	208.6	207.4
GE (MJ/kg)	17.00	16.89	18.81	18.83
Total phosphorus	8.0	8.5	8.1	7.9
Phytase activity, FTU/kg ^6^	BDL ^7^	≥1000	BDL	≥1000
Calculated crude fat content	32.8	32.8	116.2	116.2

^1^ Melojel (National Starch and Chemical Co., Bridgewater, NJ, USA). ^2^ Cerelose (Ingredion, Westchester, IL, USA). ^3^ Providing the following per kilogram of diet: vitamin A, 8250 IU; vitamin D_3_, 825 IU; vitamin E, 40 IU; niacin, 35 mg; D-pantothenic acid, 15 mg; riboflavin, 5 mg; menadione, 4 mg; folic acid, 2 mg; thiamine, 1 mg; D-biotin 0.2 mg; and vitamin B_12_, 0.025 mg. ^4^ Providing the following per kilogram of diet: Zn, 100 mg as ZnSO_4_; Fe, 80 mg as FeSO_4_; Cu, 50 mg as CuSO_4_; Mn, 25 mg as MnSO_4_; I, 0.5 mg as Ca(IO_3_)_2_; and Se, 0.1 mg as Na_2_SeO_3_. ^5^ Ronozyme HiPhos, 6-phytase, derived from *Aspergillus oryzae* (DSM Nutritional Products, Basel, Switzerland), with phytase activity at 13,750 FTU/g. ^6^ Results from enzyme analysis, which was carried by Analytical Research Center, DSM Nutritional Products Ltd., Kaiseraugst, Switzerland. ^7^ BDL = Below detection limit.

**Table 2:** Analyzed nutrient content (g/kg) and gross energy (GE) value of canola meal and canola cake included in the experimental diets (as-is basis).

Item	Canola Meal	Canola Cake
Moisture	100.0	73.5
Crude protein	402.1	368.6
Crude fat	35.0	202.0
Total phosphorus	11.5	10.2
GE (MJ/kg)	17.98	21.73

**Table 3:** Coefficients of apparent ileal digestibility (CAIDs) of nutrients and gross energy in diets containing canola meal or canola cake with or without phytase supplementation.

CAID ^2^	Canola Meal		Canola Cake		SEM ^1^	*p*-Value		
	− Phytase	+ Phytase	− Phytase	+ Phytase		Co-Product	Phytase	Co-Product × Phytase
Dry matter	0.654	0.646	0.715	0.729	0.020	<0.001	0.774	0.485
Gross energy	0.679	0.670	0.750	0.758	0.017	<0.001	0.948	0.532
Crude protein	0.683	0.680	0.725	0.748	0.016	<0.001	0.290	0.329
Phosphorus	0.258	0.414	0.275	0.483	0.048	0.320	<0.001	0.491

^1^ SEM = standard error of the mean; − Phytase = without phytase; + Phytase = with phytase. Least square means based on 5–7 pig observations per diet. ^2^ The CAID of dry matter, gross energy, crude protein and phosphorus were calculated using the index method [28].

**Table 4:** Coefficients of apparent total tract digestibility (CATTDs) of nutrients and gross energy in diets containing canola meal or canola cake with or without phytase supplementation.

CATTD ^2^	Canola Meal		Canola Cake		SEM ^1^	*p*-Value		
	− Phytase	+ Phytase	− Phytase	+ Phytase		Co-Product	Phytase	Co-Product × Phytase
Dry matter	0.809	0.808	0.839	0.842	0.007	<0.001	0.788	0.727
Gross energy	0.811	0.805	0.849	0.843	0.007	<0.001	0.226	0.994
Crude protein	0.787	0.788	0.824	0.821	0.008	<0.001	0.901	0.710
Phosphorus	0.353	0.515	0.309	0.486	0.032	0.143	<0.001	0.775

^1^ SEM = standard error of the mean; − Phytase = without phytase; + Phytase = with phytase. Least square means based on 6–7 pig observations per diet. ^2^ The CATTD of dry matter, gross energy, crude protein and phosphorus were calculated using the index method [28].

**Table 5:** Coefficients of apparent hindgut fermentation (CAHFs) of nutrients and gross energy in diets containing canola meal or canola cake with or without phytase supplementation.

CAHF ^2^	Canola Meal		Canola Cake		SEM ^1^	*p*-Value		
	− Phytase	+ Phytase	− Phytase	+ Phytase		Co-Product	Phytase	Co-Product × Phytase
Dry matter	0.155	0.162	0.124	0.113	0.018	0.008	0.883	0.508
Gross energy	0.133	0.137	0.098	0.086	0.016	0.002	0.706	0.501
Crude protein	0.115	0.114	0.092	0.075	0.017	0.026	0.469	0.513
Phosphorus	0.101	0.117	0.039	−0.022	0.057	0.030	0.590	0.352

^1^ SEM = standard error of the mean; − Phytase = without phytase; + Phytase = with phytase. Least square means based on 4–7 pig observations per diet. ^2^ The CAHF of dry matter, gross energy, crude protein and phosphorus were calculated as the coefficient of apparent total tract digestibility minus the coefficient of apparent ileal digestibility for each variable [29].

## Data Availability

The data presented in this study are available on request from the corresponding author.
